# BGLAP is expressed in pancreatic cancer cells and increases their growth and invasion

**DOI:** 10.1186/1476-4598-6-83

**Published:** 2007-12-28

**Authors:** Hany Kayed, Sandor Bekasi, Shereen Keleg, Christoph W Michalski, Thomas Giese, Helmut Friess, Jörg Kleeff

**Affiliations:** 1 Department of General Surgery, University of Heidelberg, Heidelberg, Germany; 2 Department of Surgery, Semmelweis University, Budapest, Hungary; 3 Department of Surgery, Technische Universität München, Munich, Germany; 4 Institute of Immunology, University of Heidelberg, Heidelberg, Germany

## Abstract

**Background:**

Bone gamma-carboxyglutamate protein (BGLAP; osteocalcin) is a small, highly conserved molecule first identified in the mineralized matrix of bone. It has been implicated in the pathophysiology of various malignancies. In this study, we analyzed the expression and role of BGLAP in the normal human pancreas, chronic pancreatitis (CP), and pancreatic ductal adenocarcinoma (PDAC) using quantitative RT-PCR, immunohistochemistry, immunocytochemistry and enzyme immunoassays, as well as cell proliferation and invasion assays. Gene silencing was carried out using specific siRNA molecules.

**Results:**

Compared to the normal pancreas, BGLAP mRNA and protein levels were not significantly different in CP and PDAC tissues. BGLAP was faintly present in the cytoplasm of normal acinar cells but was strongly expressed in the cytoplasm and nuclei of tubular complexes and PanIN lesions of CP and PDAC tissues. Furthermore, BGLAP expression was found in the cancer cells in PDAC tissues as well as in 4 cultured pancreatic cancer cell lines. TNFalpha reduced BGLAP mRNA and protein expression levels in pancreatic cancer cell lines. In addition, BGLAP silencing led to reduction of both cell growth and invasion in those cells.

**Conclusion:**

BGLAP is expressed in pancreatic cancer cells, where it potentially increases pancreatic cancer cell growth and invasion through autocrine and/or paracrine mechanisms.

## Introduction

Bone gamma-carboxyglutamic acid protein (BGLAP or osteocalcin) is a small, highly conserved molecule associated with mineralization of bone matrix [1]. BGLAP is an 11 kDa protein which is synthesized and secreted by normal maturing osteoblasts [2]. It regulates the dynamics of new bone formation and bone resorption [3-5] by interaction with vitamin D, and by influencing the differentiation of osteoblasts [6-8]. BGLAP is also involved in the posttranslational targeting of vitamin K-dependent gamma-carboxylation [1], which controls blood coagulation. Accordingly, defects in BGLAP expression lead to the development of chondrodysplasia punctata, coagulation defects, and coumarin embryopathy [9]. Since the discovery of BGLAP secretion in a subset of osteosarcoma cell lines [10], BGLAP has been implicated in the development of various malignant tumors. In multiple myeloma, BGLAP is considered a biochemical marker for bone resorption and dynamics during the malignant process. Thus, serum BGLAP levels are reduced in patients with multiple myeloma with osteolytic bone lesions [11]. There is also growing evidence that markers of bone metabolism correlate with the risk of skeletal complications, disease progression and tumor growth [12,13]. In prostate cancer, BGLAP is expressed in the cancer cells and improves adhesion, proliferation, and survival of tumor cells metastasizing to the bone [14]. In breast cancer, BGLAP serum levels determine the progress of the disease, especially with respect to bone metastases [15,16]. Although metastasis of pancreatic cancer to the bones is exceedingly rare, both PDAC and CP are characterized by a dense desmoplastic reaction, which in the case of CP often leads to calcification. Therefore, in the present study we analyzed the expression of BGLAP in normal and diseased human pancreatic tissues.

## Results

**Figure 1:**
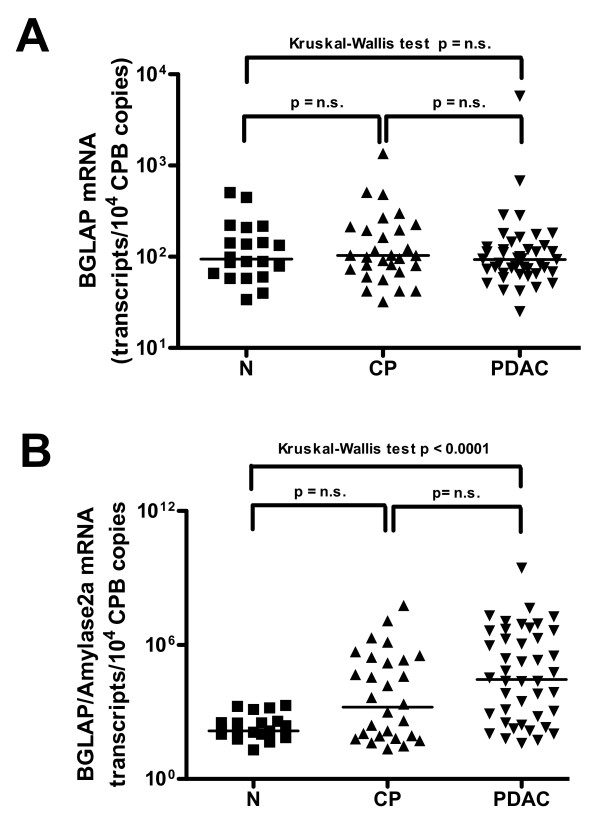
**Expression of BGLAP in pancreatic tissues**. Quantitative RT-PCR analysis of mRNA levels for BGLAP in normal pancreatic, CP and PDAC tissue samples was carried out as described in the Methods section before (A) and after (B) normalization to Amy2A. RNA input was normalized to the average expression of the two housekeeping genes HPRT and cyclophilin B, and is presented as adjusted transcripts/10,000 CPB copies.

**Figure 2:**
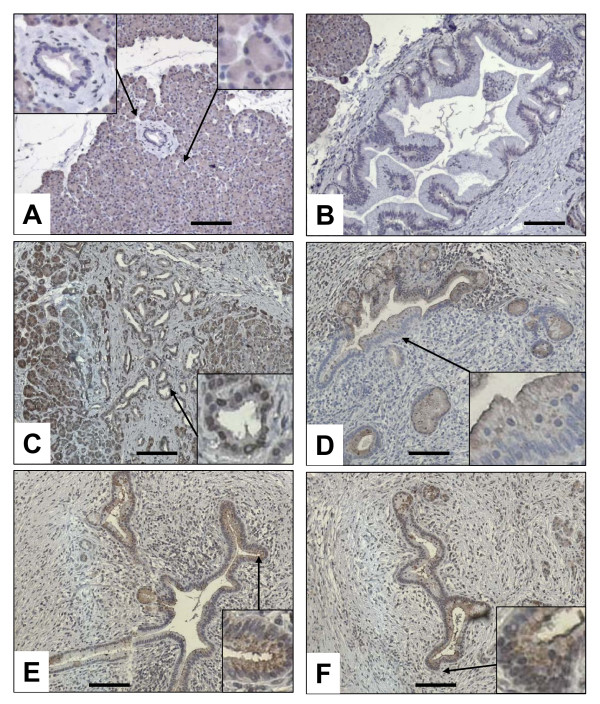
**Localization of BGLAP in normal pancreatic and CP tissues by immunohistochemistry**. Immunohistochemistry was performed using a specific BGLAP antibody as described in the Methods section. BGLAP was localized in the cytoplasm of the normal acinar cells but not in the normal ducts (A), and weakly localized in the PanIN1-2 lesions (B). In CP tissues, BGLAP was localized in the cytoplasm of the cells of the tubular complexes (C), PanIN1-2 lesions (D) and large ducts (E & F). Insets show high magnification of structures indicated by arrows. A magnification scale bar of 100 μm is shown.

**Figure 3:**
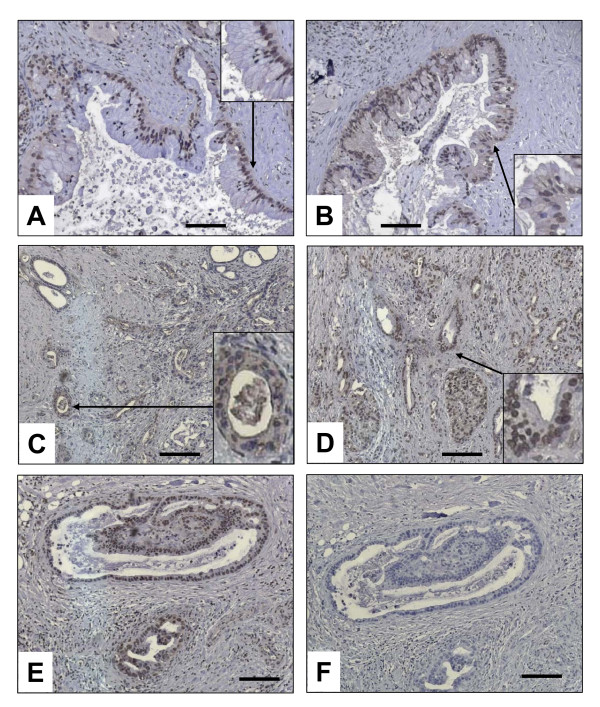
**Localization of BGLAP in PDAC tissues by immunohistochemistry**. Immunohistochemistry was performed using a specific BGLAP antibody as described in the Methods section. BGLAP exhibited a cytoplasmic and occasionally nuclear localization high-grade PanIN lesions (A & B) and cancer cells (C-E). The specificity of the staining was performed using control IgG (F). Insets show high magnification of structures indicated by arrows. A magnification scale bar of 100 μm is shown.

To exactly quantify mRNA levels of BGLAP in bulk pancreatic tissues, qRT-PCR was carried out. This analysis demonstrated no significant difference between median BGLAP mRNA levels in the normal pancreas, CP and PDAC tissues (figure 1A). As the cellular composition of bulk PDAC and CP tissues is different than that of normal pancreatic tissues, BGLAP mRNA values were normalized to amylase-2A (Amy2A) mRNA levels for each tissue sample to exclude the mRNA expression of BGLAP in the acini (figure 1B). This analysis revealed a significant increase in the mRNA ratio of BGLAP/Amy2A in PDAC (p < 0.0001) compared to normal pancreatic tissues. This suggests that in PDAC, tissue elements other than acini contribute to the observed BGLAP mRNA levels in bulk tissues. Therefore, and in order to localize BGLAP, immunohistochemistry was performed on pancreatic tissue sections from normal (n = 10), CP (n = 20) and PDAC (n = 20) cases. BGLAP expression was weak to absent in normal pancreatic ductal cells of 9/10 normal pancreatic tissues (figure 2A). In contrast, acinar cells of 9/10 normal pancreatic tissues exhibited moderate cytoplasmic BGLAP staining (figure 2A). PanIN1-2 lesions in normal pancreatic tissues demonstrated weak to moderate BGLAP staining (figure 2B). In contrast, in CP tissues there was moderate cytoplasmic and occasionally nuclear staining of tubular complexes in 18 out of 20 cases (figure 2C). Moderate BGLAP staining was also observed in the PanIN1-2 lesions (figure 2D), as well as in ductal cells (figure 2E &2F). In 15/20 PDAC tissues, moderate to strong cytoplasmic and occasionally nuclear BGLAP staining was observed in the tubular complexes (figure 3A &3B), PanIN1-3 lesions (figure 3C &3D), and cancer cells (figure 3E). The specificity of the staining was confirmed using normal mouse IgG as a negative control in consecutive sections (figure 3F).

**Figure 4:**
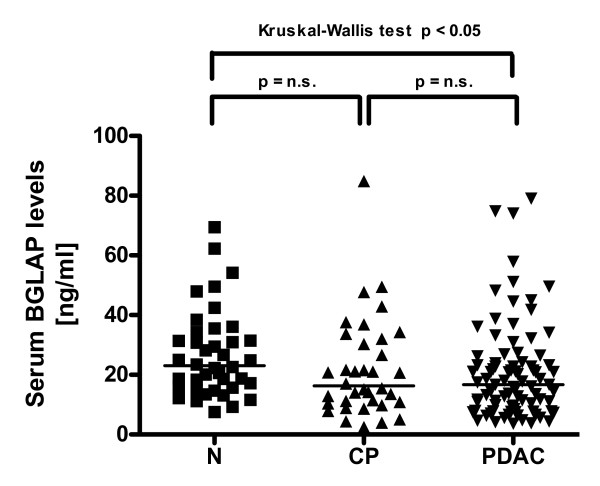
**Enyzme immunoassay of tissue samples**. Enzyme immunoassay of BGLAP levels in normal pancreatic, CP and PDAC tissue samples was carried out as described in the Methods section. Horizontal bars represent median values.

Next, BGLAP serum levels were determined in CP and PDAC patients and compared to healthy volunteers. This analysis revealed a significant reduction in BGLAP serum levels in PDAC patients (figure 4; p < 0.05). There was no significant difference between CP and PDAC serum BGLAP levels. Correlation analysis of BGLAP mRNA and serum levels with clinico-pathological parameters of PDAC patients revealed no significant difference in the median BGLAP mRNA or serum levels in PDAC patients with lymph node metastasis compared to PDAC patients without lymph node metastasis (p = 0.9; data not shown). There was also no correlation with other parameters such as tumor size or grade.

**Figure 5:**
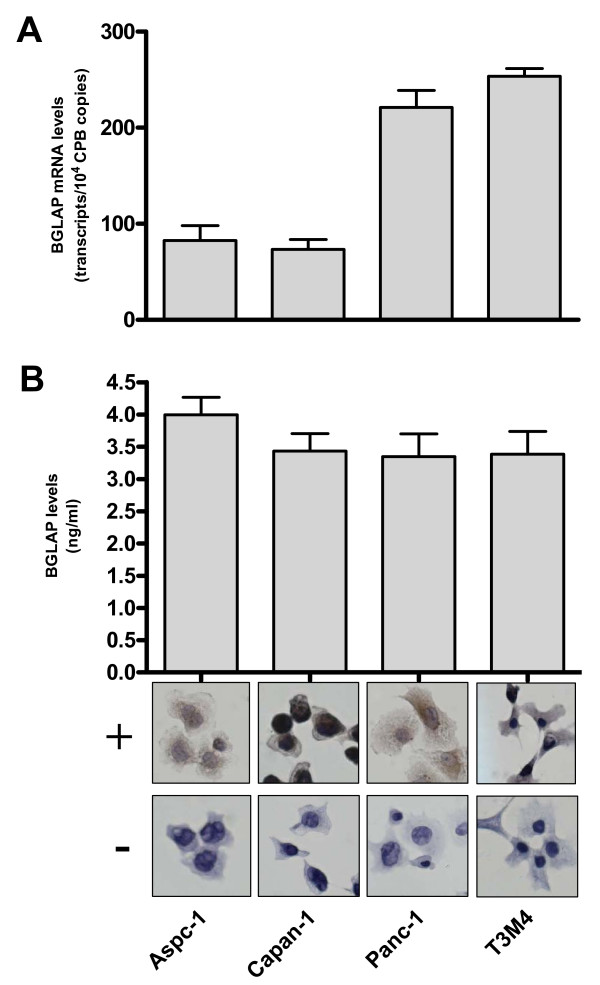
**Expression and localization of BGLAP in four cultured pancreatic cancer cell lines**. (A) QRT-PCR analysis of mRNA levels for BGLAP in pancreatic cancer cells was carried out as described in the Methods section. RNA input was normalized to the average expression of the two housekeeping genes HPRT and cyclophilin B. Bars represent the mean values +/- SEM of 3 independent experiments as adjusted transcripts/10,000 CPB copies. (B) Enzyme immunoassay of BGLAP levels in cell culture supernatant of pancreatic cancer cell lines was carried out as described in the Methods section. Bars represent the mean values +/- SEM of 3 independent experiments in ng/ml. Localization of BGLAP in the cultured pancreatic cancer cell lines was performed by immunocytochemistry as described in the Methods section, using a specific BGLAP antibody (+). The specificity of the staining was confirmed using control IgG (-).

In a subsequent step, the expression of BGLAP was determined in Aspc-1, Capan-1, Panc-1 and T3M4 cultured pancreatic cancer cells. BGLAP mRNA was detected in these cell lines with a range of 73–254 copies/10,000 CPB copies (figure 5A). In addition, BGLAP protein was detected in the cell culture supernatants of the same four cell lines by EIA, where the range of BGLAP expression was 3.4 to 4 ng/ml (figure 5B). BGLAP was present in the cytoplasm and occasionally in the nuclei of pancreatic cancer cell lines as determined in the four cultured pancreatic cancer cell lines by immunocytochemistry (figure 5B).

**Figure 6:**
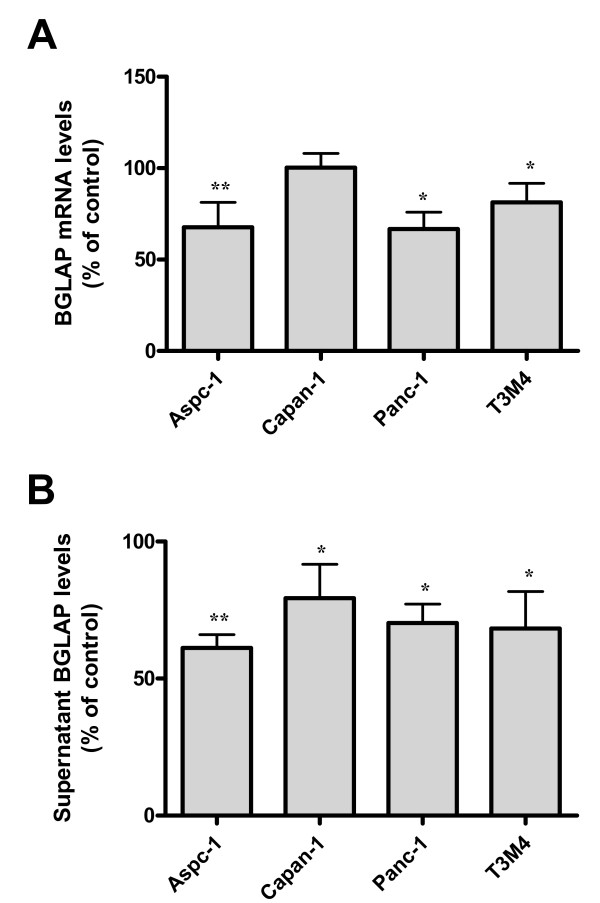
**Effects of TNF-α on BGLAP expression in pancreatic cancer cells**. (A) Pancreatic cancer cells were treated with the indicated dose of TNF-α for 48 h. Cell lysates and cell culture supernatants were collected for detection of BGLAP mRNA and protein expression, respectively, as described in the Methods section. Bars represent BGLAP mRNA (A) and protein expression levels (B) as a percentage of untreated cells and as determined by QRT-PCR and enzyme immunoassay, respectively. Data are presented as mean +/- SEM of three independent experiments (*: p < 0.05, **: p < 0.01).

Since BGLAP was expressed in pancreatic cancer tissues and cells, various growth factors that might be involved in pancreatic carcinogenesis were examined for their effect on BGLAP expression. Aspc-1, Capan-1, Panc-1 and T3M4 pancreatic cancer cells were treated with recombinant TGF-β1, BMP2, FGF2, Shh, Ihh and TNF-α for 48 h as described previously [17]. Of those cytokines, only TNF-α was able to moderately but consistently reduce BGLAP mRNA and protein levels in the four cultured pancreatic cancer cell lines (figure 6A &6B). The maximum effects were observed in Aspc-1, where BGLAP mRNA and protein levels were reduced by -32.3 +/- 7.9% and -38.8 +/- 2% (p < 0.01), respectively. The other cytokines demonstrated no significant effects on BGLAP expression in the tested pancreatic cancer cells (data not shown).

**Figure 7:**
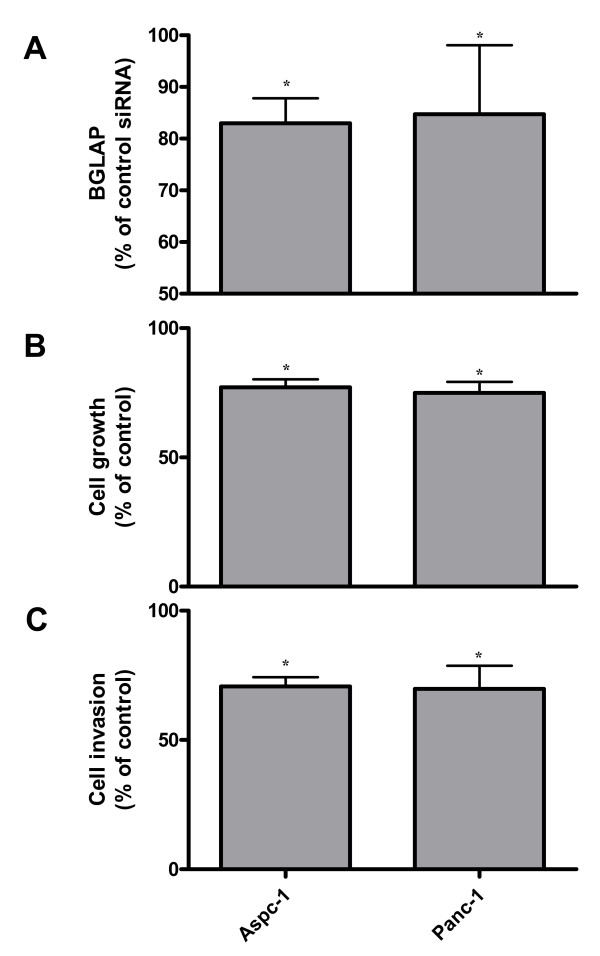
**The effects of BGLAP silencing on pancreatic cancer cell growth and invasion**. (A) Aspc-1 and Panc-1 pancreatic cancer cells were subjected to BGLAP silencing using two different specific BGLAP siRNA molecules, and the effects of silencing were measured by enzyme immunoassay as described in the Methods section. (B) Cell growth and (C) invasion assays were performed as described in the Methods section. Data are presented as mean +/- SEM compared to the respective controls of three independent experiments (*: p < 0.05).

In order to determine the role of BGLAP in regulating the growth and invasion of pancreatic cancer cells, BGLAP was silenced using siRNA molecules in Aspc-1 and Panc-1 pancreatic cancer cells. BGLAP silencing effects (-17 +/- 2.3% and -15.3 +/- 6.3%) (p < 0.05) were detected by EIA in cell culture supernatant of both Aspc-1 and Panc-1 cells, respectively (figure 7A). After BGLAP silencing, cell growth and invasion assays were carried out. Both Aspc-1 and Panc-1 cells exhibited a reduction in cell growth (-22.8 +/- 1.8% and -25.1 +/- 2.4% [p < 0.05], respectively) compared to cells transfected with control siRNA (figure 7B). In addition, both Aspc-1 and Panc-1 cells exhibited a reduction in cell invasion (-29.3 +/- 1.5% and -30.2 +/- 3% [p < 0.05], respectively) compared to cells transfected with control siRNA (figure 7C).

## Discussion

BGLAP is a small protein (10.9 kDa) [2] that is secreted by osteosarcoma cells [10] and is involved in bone turn-over of metastatic lesions from tumors such as prostate and breast cancers. In the present study, BGLAP mRNA levels were not significantly different between bulk normal pancreatic, CP and PDAC tissues. Normal pancreatic tissues consist mainly of acinar cells (and to a lesser extent of islets and ductal cells). In contrast, in CP tissues, the acinar cell compartment is replaced by extensive fibrosis, ductal proliferation, and acinar cell conversion into tubular complexes [18-21]. These changes further progress towards malignant transformation and cancer cell proliferation in PDAC [19,22]. In the present study, BGLAP was localized in the acini of normal pancreatic tissues, indicating that the mRNA content of BGLAP represents the acinar fraction in normal pancreatic tissues. Interestingly, BGLAP was localized in the tubular complexes and PanIN lesions of both CP and PDAC as well as in the cancer cells in PDAC. This was indirectly confirmed by normalizing BGLAP mRNA levels to the acinar marker Amy2A. Using this method, a significant increase in the ratio of BGLAP/Amy2A mRNA expression in PDAC tissues was observed compared to normal pancreatic tissues. This might indicate that BGLAP is expressed in the disease-specific tissue elements such as the tubular complexes, PanINs and cancer cells. Surprisingly, there was a decrease of BGLAP serum levels in PDAC patients compared to healthy controls. These findings suggest that BGLAP is principally released by acinar cells in the normal and diseased pancreas. However, since cytoplasmic expression of BGLAP was also observed in tubular complexes and cancer cells, it could be speculated that BGLAP is not efficiently released from these cells. This might explain the elevated BGLAP serum levels in healthy controls despite similar amounts of BGLAP mRNA in bulk diseased pancreatic tissues.

Numerous growth factors and cytokines – such as TGF-β1 [23,24], BMP2 [25], FGF2 [26], Shh [27], Ihh [28] and TNF-α [29] – have been implicated in the pathogenesis of PDAC. Among those factors, only TNF-α reduced the expression of BGLAP in pancreatic cancer cell lines. The other growth factors and cytokines exhibited no effects on BGLAP expression in the tested pancreatic cancer cells. TNF-α has anti-tumor effects in pancreatic adenocarcinoma [29-31]. The down-regulation of BGLAP by TNF-α in pancreatic cancer cells indicates that BGLAP is one of the TNF-α targets and indirectly suggests a tumor-promoting function of BGLAP in pancreatic cancer. In line with this argument, down-regulation of BGLAP levels using siRNA molecules resulted in a significant reduction in pancreatic cancer cell growth and invasion. This has also been shown in other tumors, such as osteosarcoma [32,33].

The transcription of BGLAP is dependent on the expression of runx2 transcription factor, which binds to an osteoblast-specific cis-acting element in the promoter of BGLAP [34]. Runx2 is over-expressed in PDAC [17], and other factors downstream of runx2, such as osteopontin [35] and SPARC [36], are also over-expressed in PDAC and have the potential to increase pancreatic cancer invasion and metastasis [37,38]. In contrast, runx2 represses the expression of bone sialoprotein [39], which is only weakly expressed in pancreatic cancer cells [39].

## Conclusion

In conclusion, BGLAP is expressed in the tubular complexes and cancer cells of CP and PDAC tissues and has the potential to increase pancreatic cancer cell growth and invasion.

## Materials and methods

### Tissue and serum sampling

Pancreatic tissue specimens were obtained from 20 CP and 20 PDAC patients with a median age of 62.5 years (range: 41–78 years) in whom pancreatic resections were performed. Normal human pancreatic tissue samples were obtained through an organ donor program from 10 previously healthy individuals (median age: 45 years; range: 18–76 years). For all samples, the diagnosis was confirmed histologically. Freshly removed tissues (within 5 min after surgical excision) were: a) fixed in paraformaldehyde solution for 12 to 24 h and then paraffin embedded for histological analysis; b) kept in RNAlater (Ambion Ltd., Huntingdon, Cambridgeshire, United Kingdom) for RNA analysis, or c) snap-frozen in liquid nitrogen and maintained at -80°C for protein analysis. Serum samples were obtained from 87 PDAC patients (median age: 63 years) and 36 CP patients (median age: 50 years). Forty serum samples were obtained from healthy volunteers (median age: 35 years). Fresh blood samples were collected and centrifuged. The serum supernatant was collected in polyethylene tubes and kept frozen at -80°C until use. The Human Ethics Committee of the University of Heidelberg, Germany, approved all studies, and written informed consent was obtained from all patients.

### Cell culture

ASPC-1, Capan-1, and T3M4 pancreatic cancer cells were routinely grown in RPMI 1640 medium (Life Technologies, Karlsruhe, Germany), supplemented with 10% FCS (Sigma-Aldrich, St. Louis, MO) and 100 U/ml penicillin/streptomycin (Invitrogen GmbH, Karlsruhe, Germany). Panc-1 was routinely grown in DMEM medium (Invitrogen), supplemented with 10% FCS and 100 U/ml penicillin/streptomycin. For induction experiments, cells were seeded in 10 cm cell culture plates in 10% FCS growth medium and allowed to attach for 12 h. Growth medium was replaced by serum-reduced medium (1% FCS), and supplemented with recombinant TGF-β1 (500 pM), BMP2 (100 ng/ml) bFGF2 (10 ng/ml), sonic hedgehog (Shh) (500 ng/ml), indian hedgehog (Ihh) (500 ng/ml) (all from R&D Systems GmbH, Wiesbaden, Germany) or TNF-α (100 ng/ml; Promega Biosciences Inc., Mannheim, Germany) for 48 h. Afterwards, cell culture supernatants and mRNA were collected as described.

### Immunohistochemistry

Immunohistochemistry was performed as previously described [40] with slight modifications. Consecutive 5 μm-thick paraffin-embedded tissue sections were deparaffinized and rehydrated in progressively decreasing concentrations of ethanol. After antigens were retrieved by boiling the tissue sections in 10 mM citrate buffer for 10 min in the microwave oven, endogenous peroxidase activity was quenched by incubation in deionized water containing 3% hydrogen peroxide at room temperature for 10 min. The slides were then washed in washing buffer (10 mM Tris-HCl, 0.85% NaCl, 0.1% bovine serum albumin, and pH 7.4), and incubated with the mouse monoclonal BGLAP antibody (ZYTOMED Systems GmbH, Berlin, Germany) diluted in a universal block reagent (DAKO Corporation, Carpentaria, CA) for 18 h at 4°C. In consecutive sections, the specificity of the primary antibody was confirmed using the corresponding normal mouse IgG (DAKO). The slides were then rinsed with washing buffer and incubated with anti-mouse HRPO-labeled IgG (Amersham International, Buckinghamshire, UK) diluted in a universal blocking reagent (DAKO) for 1 h at room temperature. The tissue sections were then washed in washing buffer and each section was subjected to 100 μl of DAB-chromogen/substrate reagent (DAKO) and counterstained with Mayer's hematoxylin.

Immunocytochemistry was performed as described previously [41]. Briefly, pancreatic cancer cells were cultured on Super Frost microscope slides (Menzel GmbH & Co KG, Braunschweig, Germany) overnight till adherent and then washed with phosphate buffered saline (PBS), fixed with 3.5% para-formaldehyde for 25 min, and quenched with 30 mM glycine/PBS for 5 min, followed by permeabilization of the cell membrane with 0.1% Triton x-100 for 5 min at room temperature. Next, slides were incubated with the mouse monoclonal BGLAP antibody and immunostaining was performed as described above. Slides were analyzed using the Axioplan 2 imaging microscope (Carl Zeiss light microscope, Göttingen, Germany).

### Enzyme immunoassay (EIA)

Determination of the BGLAP protein levels was performed with an enzyme immunoassay (EIA) kit (Takara Bio Inc., Shiga, Japan). Briefly, 96-well flat-bottomed plates pre-coated with the mouse monoclonal BGLAP antibody were loaded with 100 μl serum, cell culture supernatant or protein standards for 2 h at room temperature. After washing with PBS, 100 μl of the BGLAP antibody labeled with peroxidase (POD) were loaded into the wells for 1 h at room temperature. Next, 100 μl of substrate solution were added to the wells for 20 min at room temperature, followed by the addition of 100 μl 1N H_2_SO_4 _stop solution. Then, absorbance was measured at 450 nm using a microtiter plate reader (Opsys MR, Thermo Labsystems, Frankfurt, Germany).

### siRNA transfection

Aspc-1 and Panc-1 pancreatic cancer cells were grown in complete RPMI or DMEM medium in 10 ml cell culture plates until 50% confluence. BGLAP siRNA transfection was performed using RNAifect transfection reagent (Qiagen, Hilden, Germany) according to the manufacturer's instructions. Cells were transfected with a 5 μg mixture of two BGLAP siRNA target sequences – Hs-BGLAP-3-HP siRNA (AAG CAG GAG GGC AGC GAG GTA) and Hs-BGLAP-4-HP siRNA (CCC AGG CGC TAC CTG TAT CAA) – as well as with a control siRNA target sequence (AAT TCT CCG AAC GTG TCA CGT) (Qiagen, Hilden, Germany) for 48 h.

### Cell growth assays

Aspc-1 and Panc-1 pancreatic cancer cells transfected with BGLAP siRNAs or control siRNA were seeded at a density of 5000 cells/well in 96-well plates for 48 h. Then, 10 ml MTT (5 mg/ml) dissolved in PBS pH 7.4 were added to each well and incubated for 4 h at 37°C. Subsequently, cellular formazan crystals were solubilized with 0.04 mM HCl/isopropanol. Optical density was measured at 570 nm with an ELISA plate reader (Opsys MR). All assays were performed in triplicate.

### In vitro invasion assays

The Matrigel invasion assay (BD Biosciences, Heidelberg, Germany) was used to assess the invasive potential of pancreatic cancer cells following BGLAP silencing. Briefly, BioCoat Matrigel invasion chambers were rehydrated according to the manufacturer's instructions. Five hundred μl of DMEM cell culture medium supplemented with 10% FCS were added to the bottom of 24-well plates. Aspc-1 and Panc-1 pancreatic cancer cells transfected with BGLAP siRNAs or control siRNA were seeded at a density of 50,000 cells/well into the upper inserts and incubated at 37°C. After 24 h, the non-invading cells were removed from the upper surface of the separating membrane by gentle scrubbing with a cotton swab. Invading cells were fixed in ice-cold 100% methanol and stained with 0.05% crystal violet in 20% ethanol. The membranes were mounted on glass slides and manually counted using a light microscope. The invasion index was calculated as the percentage of invading cells in the treatment group compared to the control group. All assays were performed in triplicate.

### Statistical analysis

For statistical analyses, the non-parametric Mann-Whitney test and the Kruskal-Wallis test (followed by Dunn's post-hoc test) were used, unless indicated otherwise. Significance was defined as p < 0.05.

## Competing interests

The author(s) declare that they have no competing interests.

## Authors' contributions

HK and SB carried out the preparation of samples, performed the experiments and molecular analyses and participated in drafting the manuscript. SK participated in performing the immunohistochemical and immunocytochemical experiments. CWM participated in drafting the manuscript and in the statistical analyses. TG carried out the quantitative RT-PCR analyses, designed the corresponding primers and evaluated the resulting data. HF and JK participated in designing the study, in performing the statistical analyses and coordinated and helped to draft the manuscript. All authors read and approved the final manuscript.
